# Design of Wearable EEG Devices Specialized for Passive Brain–Computer Interface Applications

**DOI:** 10.3390/s20164572

**Published:** 2020-08-14

**Authors:** Seonghun Park, Chang-Hee Han, Chang-Hwan Im

**Affiliations:** Department of Biomedical Engineering, Hanyang University, Seoul 04763, Korea; qqzzwwxx12@hanyang.ac.kr (S.P.); zeros8706@naver.com (C.-H.H.)

**Keywords:** wearable EEG device, passive brain–computer interface, electroencephalography, affective computing

## Abstract

Owing to the increased public interest in passive brain–computer interface (pBCI) applications, many wearable devices for capturing electroencephalogram (EEG) signals in daily life have recently been released on the market. However, there exists no well-established criterion to determine the electrode configuration for such devices. Herein, an overall procedure is proposed to determine the optimal electrode configurations of wearable EEG devices that yield the optimal performance for intended pBCI applications. We utilized two EEG datasets recorded in different experiments designed to modulate emotional or attentional states. Emotion-specialized EEG headsets were designed to maximize the accuracy of classification of different emotional states using the emotion-associated EEG dataset, and attention-specialized EEG headsets were designed to maximize the temporal correlation between the EEG index and the behavioral attention index. General purpose electrode configurations were designed to maximize the overall performance in both applications for different numbers of electrodes (2, 4, 6, and 8). The performance was then compared with that of existing wearable EEG devices. Simulations indicated that the proposed electrode configurations allowed for more accurate estimation of the users’ emotional and attentional states than the conventional electrode configurations, suggesting that wearable EEG devices should be designed according to the well-established EEG datasets associated with the target pBCI applications.

## 1. Introduction

In the past decades, brain–computer interfaces (BCIs) based on electroencephalograms (EEGs) have been intensively studied for helping disabled people communicate with the external environment [1]. Various BCI applications have been developed for these people, including mental spellers [2], patient-assistant systems [3], neuro-rehabilitation [4], and external device control [5], demonstrating the potential of EEG-based BCIs as practical assistive tools.

Recently, with the growing public interest in BCI technology, BCIs have also been developed for healthy people. In particular, the rapid advances in system-on-a-chip (SoC) technology have facilitated the release of wearable EEG devices that can be employed for commercial BCI applications for healthy individuals. For example, InteraXon Inc. (Toronto, ON, Canada) launched Muse^TM^, which is a headband-type wearable EEG device that assists meditation. Other examples include Melomind^TM^ (myBrain Technologies Inc., Paris, France), BrainBit^TM^ (BrainBit Inc., Brooklyn, NY, USA), and Diadem^TM^ (Bitbrain Inc., Zaragoza, Spain). While these wearable devices still have limitations in their relatively low signal quality [6,7], they present several advantages over the conventional wired, bulky EEG devices in that they are economical, portable, and easy to use [8]. Generally, these portable EEG devices have been employed for passive BCI (pBCI) applications. This emerging category of BCIs aims to decode the user’s mental states, such as emotions, mental workload, and attention [9,10]. pBCI technology has been used in various applications, such as neuromarketing [11,12], neurocinematics [13], neuroeducation [14], sleep monitoring [15], sensorial evaluation [16], driver vigilance detection [17], and human factor evaluation [18,19,20].

The wearable EEG devices currently available on the market can be classified into headband- and headset-type devices. The headband-type devices measure the EEG signals from the non-hair bearing area (i.e., forehead). These devices are easy to set up, aesthetically pleasing, and efficient because they measure the EEG signals from the forehead over the prefrontal cortex, which is associated with various human mental states, such as attention, emotion, and cognition [21,22]. In contrast, EEG signals recorded around the prefrontal cortex are prone to contamination by artifacts, such as electrooculogram (EOG) and electromyogram artifacts [23]. Furthermore, these devices cannot be used for some applications, owing to the limited coverage of the sensors.

The headset-type EEG devices can measure EEG signals from much wider brain areas than those covered by the headband-type devices: not only from the prefrontal cortex but also from other brain areas, including the parietal, temporal, and occipital cortices. EEG signals recorded from wider brain areas allow the BCI system to estimate various mental states with higher accuracy; thus, headset-type EEG devices can be employed in a wider range of applications when compared to headband-type EEG devices. For instance, the global field synchronization—a measure calculated using EEG signals acquired from the entire brain—was reported to be effective for estimating users’ emotional valence [24]. Additionally, Clerico et al. reported that the use of connectivity features between multiple interhemispheric electrode pairs could enhance the overall accuracy of emotion classification [25]. However, a cumbersome preparation process is generally involved for appropriately wearing the devices, as the electrodes must remain in tight contact with the scalp surface in the hair-bearing area [26].

In general, wearable EEG devices employ their own electrode configurations. However, no studies have been performed on the suitability of the electrode configurations for the intended pBCI applications. In many cases, it is unclear as to how the electrode configurations of the commercial wearable EEG systems were determined. Herein, we present a detailed procedure for designing wearable EEG devices that can maximize the performance for desired BCI applications by utilizing EEG databases associated with these applications. We employed EEG datasets collected in two different studies regarding emotion classification and attention estimation because these are the two BCI applications for which wearable EEG devices are most frequently developed. We first designed two electrode configurations, each of which was optimized for each designated application. We then designed general purpose EEG electrode configurations with 2, 4, 6, and 8 electrodes, which maximized the overall performance for both applications. Finally, the classification and estimation performances achieved with the optimized electrode configurations were compared with those achieved with electrode configurations of commercial wearable EEG devices with the same number of electrodes.

## 2. Materials and Methods

In this study, we first designed emotion-specialized EEG headsets that maximize the accuracy of classifying different emotional states using an emotion-associated EEG dataset (a dataset open to the public). Subsequently, we designed attention-specialized EEG headsets that maximize the temporal correlation between the EEG index and the behavioral attention index using an attention-associated EEG dataset (a dataset that we collected in a previous study). Finally, general purpose electrode configurations were designed to maximize the overall performance in both applications, for different numbers of electrodes (2, 4, 6, and 8 electrodes). The detailed study procedures are described in the following sections.

### 2.1. Emotion Dataset and Data Analysis

#### 2.1.1. Emotion Dataset

The database for emotion analysis using physiological signals (DEAP) [27] was employed as the emotion dataset in the present study. A total of 32 healthy subjects (females: 16, age: 19–37 years) participated in the experiment for constructing the database. No one experienced an attention deficit, neurological syndromes, or any psychiatric syndromes that might affect the experimental outcomes. All participants had normal or corrected-to-normal vision. All of them were right-handed except for one female subject. They watched 40 1-min-long music videos that were edited to elicit four different emotions defined based on the two-dimensional valence-arousal model [28]: high arousal–high valence (HAHV), high arousal–low valence (HALV), low arousal–high valence (LAHV), and low arousal–low valence (LALV). The EEG data were recorded from 32 channels attached to the scalp surface according to the modified 10–20 system with an ActiveTwo EEG acquisition system (Biosemi, Amsterdam, The Netherlands) at a sampling rate of 512 Hz. Although the DEAP database included other physiological signals, such as the galvanic skin response, photoplethysmography, and body temperature, only the EEG data were used for further analyses.

#### 2.1.2. Preprocessing

**Figure 1:**
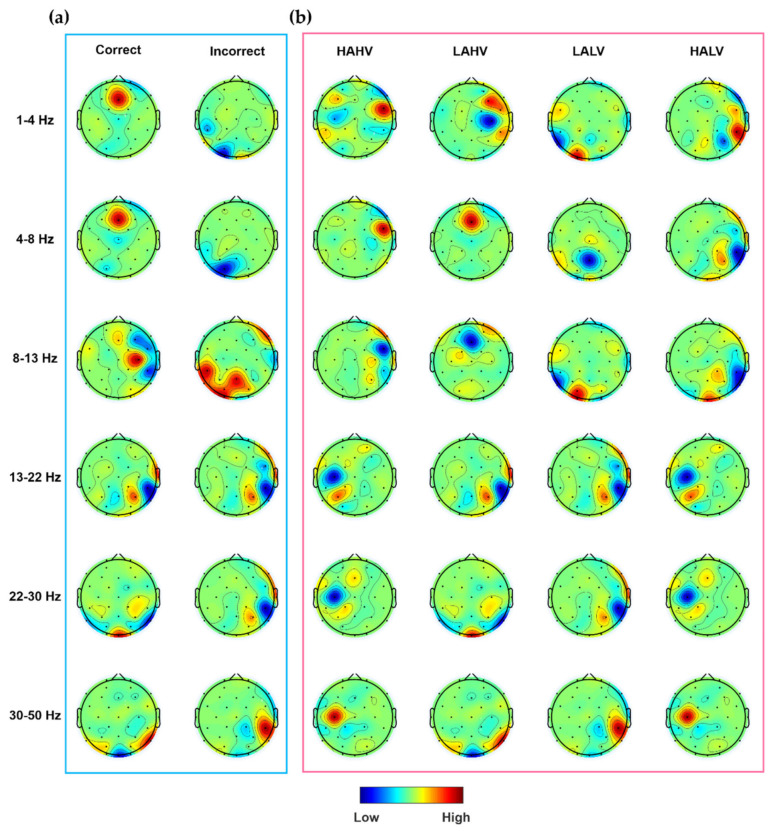
The topographic distributions of the filter-bank spatial filters derived from (**a**) subject 11 in the attention dataset and (**b**) subject 14 in the emotion dataset. Correct/incorrect represents task response correctness and HAHV/LAHV/LALV/HALV represent four emotional classes.

The raw EEG data were re-referenced to the common average reference and baseline-corrected by subtracting the average of the signal per channel. Then, to remove low frequency drift and power line noise (60 Hz), bandpass filtered at cut-off frequencies of 1 and 55 Hz using a 6th-order zero-phase Butterworth infinite impulse response filter implemented in MATLAB (MathWorks, Inc., Natick, MA, USA). For ocular artifacts rejection, we segmented the data into epochs with a 1 s moving window having a 50% overlap, yielding total 4760 epochs for each participant. Then, the epochs containing eye blinks detected by multi-window summation of derivatives within a window (MSDW) algorithm [29] were rejected, instead of removing the artifacts to clean the data. Eight subjects whose number of rejected epochs exceeded half of the total number of epochs were excluded from the further analyses. The average ratio of the number of remaining epochs to the total number of epochs was 75.39%. To further verify the effectiveness of the eye-blink artifact rejection procedure employed in this study, we presented the topographic distribution of the CSP pattern of some participants in Figure 1. Note that the values at each electrode location represent the weights of spatial filters computed in Section 2.1.3 and Section 2.2.2 after the eye-blink rejection. The topographic maps did not exhibit typical frontal lateralized EOG patterns, implying that the EOG rejection process was effective.

#### 2.1.3. Feature Extraction

Power spectral density (PSD), differential asymmetry (DASM), rational asymmetry (RASM), Hjorth parameters (HP), Shannon entropy (SE), Hurst exponent (HE), Kolmogorov complexity (KC), higher-order cumulants (HOC), and common spatial pattern (CSP) were evaluated as the features for the emotion classification. The equations to compute the features are presented in Table 1. Note that the spectral features (i.e., PSD, DASM, and RASM) and CSP were calculated for each of the following six sub-frequency bands: delta (1–4 Hz), theta (4–8 Hz), alpha (8–13 Hz), low beta (13–22 Hz), high beta (22–30 Hz), and gamma (30–50 Hz). Thus, when all 32 channels were used, the total number of EEG spectral features was [6 (PSD) × 32 (channels) + 6 (DASM) × 14 (pairs) + 6 (RASM) × 14 (pairs) + (3 (HP) + 1 (SE) + 1 (HE) + 1 (KC) + 1 (HOC)) × 32 (channels) + 12 (CSP) × 4 (emotion classes)] = 632.

#### 2.1.4. Feature Selection and Classification

The optimal feature subset was determined according to the Fisher score, i.e., the ratio of the interclass variance to the intraclass variance. The maximum number of selected features was set as 20 to avoid potential overfitting. A support vector machine (SVM) was employed as the classification model, and the SVM classifier was modeled using a linear SVM with default parameter (C = 1), implemented with a fitcecoc() function in MATLAB 2017b. To compare the results with other classifiers, we additionally applied linear discriminant analysis (LDA) and decision tree (DT) models to the same dataset. The LDA and DT classifiers were modeled with default parameter (C = 1), using fitcdiscr() and fitctree() functions in MATLAB 2017b, respectively. However, because the overall performance of SVM was better than those of LDA and DT, we only presented the SVM results in this manuscript and presented the other results as Supplementary Materials.

**Figure 2:**
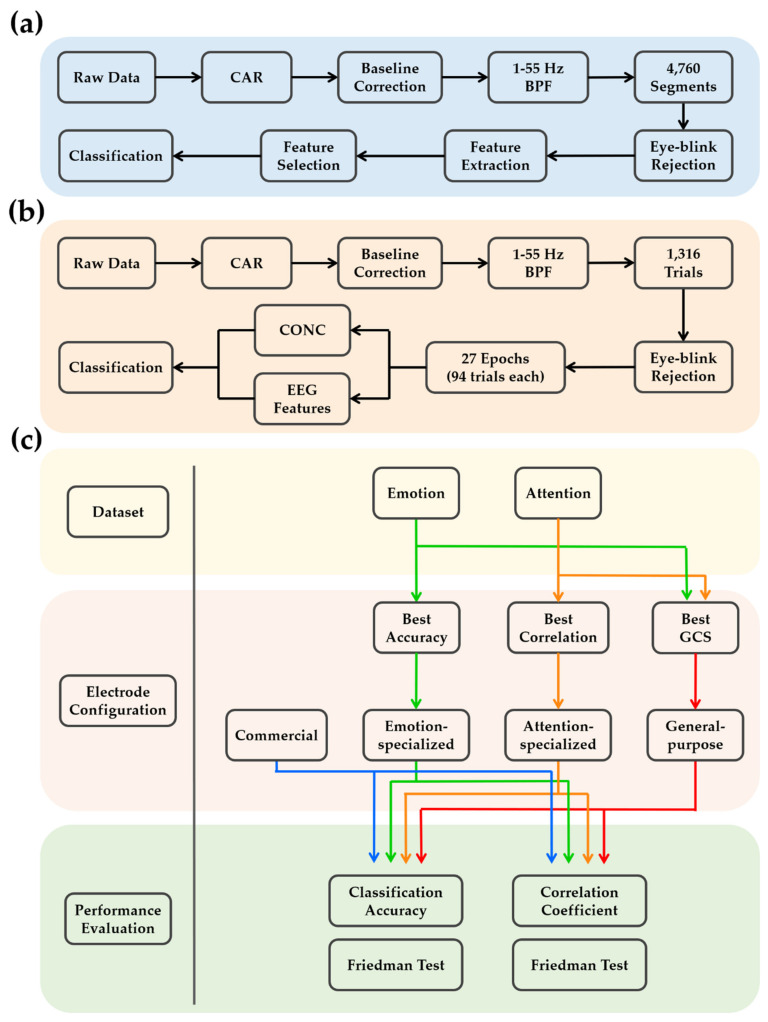
The schematic diagrams of the data analysis procedure. (**a**) Procedure for the emotion dataset analysis. (**b**) Procedure for the attention dataset analysis. (**c**) The procedure to determine and evaluate the application-specialized and general purpose electrode configurations.

We classified 40 trials into four emotions in the valence–arousal dimensions, assigning 10 trials in each quadrant (i.e., HAHV, HALV, LAHV, and LALV). A 10 × 10-fold cross-validation scheme was employed to evaluate the average classification accuracy. This procedure randomly divides the dataset into ten equal-sized partitions. Nine partitions were used for training the classifier and the remaining partition was used for testing the performance of the classifier. This procedure was repeated ten times and the final classification accuracy was calculated by averaging the ten separate results. The overall procedure of analyzing emotion dataset is summarized in Figure 2a.

### 2.2. Attention Dataset and Data Analysis

#### 2.2.1. Attention Dataset

A widely used human attention test paradigm called “d2-test of attention” [36] was employed in the experiment. In this paradigm, characters with dots were presented on a computer screen, and the participants were instructed to discriminate target characters (“d” with two dots) from distractors (“d” with a single dot or “p” with a single dot or two dots) by pressing designated keyboard buttons as quickly as possible. The behavioral performance in this task reflects mental concentration [37,38]. A total of 31 healthy university students (females: 18, age: 20–25 years) participated in the experiment. Any participant with mental retardation, head injury with a loss of consciousness, or any other neurological disorders that might have affected the experiment were excluded. All participants had normal or corrected vision, and all of them were right-handed. The participants performed the task repeatedly for 1316 trials while 32-channel EEG and horizontal and vertical EOG signals were recorded. The EEG electrode locations and the signal-acquisition system were exactly the same as those of the emotion dataset. The experiment lasted approximately 12 min on average. All experiments were performed between 3 pm and 5 pm of each day to avoid potential diurnal effects. The participants were asked not to intake any caffeine or alcohol on the day of experiment. Details regarding the study participants and experimental procedure have been presented in our previous work, which involved the same dataset [38].

#### 2.2.2. Data Analysis

The preprocessing procedure was same as that applied to the emotion dataset. For ocular artifacts rejection, the trials containing eye blinks detected by the MSDW algorithm [29] were rejected, instead of removing the artifacts to clean the data. Six subjects whose number of rejected trials exceeded half of the total number of trials were excluded from the further analyses. The average ratio of the number of remaining trials to the total number of trials was 71.70%. We segmented the data into epochs with a sliding window containing 94 trials with a 50% overlap, resulting in a total of 27 epochs. For each epoch, we calculated the same EEG features evaluated for the emotion dataset (i.e., PSD, DASM, RASM, HP, SE, HE, KC, HOC, and CSP introduced in Section 2.1.3). For each epoch, we calculated a behavioral measure called the normalized concentration performance (CONC) [37], which reflects the instantaneous attentional state. The CONC was calculated as follows:

(1) $$\mathrm{CONC}=\frac{NCT-NIT}{NTT},$$

where *NCT* represents the total number of correctly discriminated targets, *NIT* represents the total number of incorrectly discriminated targets, and *NTT* represents the total number of targets. Successive evaluation of CONC values over all epochs allowed for the estimation of attention changes over time. The EEG features evaluated for the emotion dataset were calculated for each epoch of the attention dataset. Then, the absolute value of the correlation coefficient between the behavioral measure (changes of CONC values over 27 epochs) and each of the EEG features (changes of feature values over 27 epochs) was calculated for each participant to evaluate the performance of each EEG feature for estimating the attentional state changes. Finally, the largest absolute correlation coefficient value was collected for each participant. The overall procedure of analyzing attention dataset is summarized in Figure 2b.

### 2.3. Determination of Optimal Electrode Configuration

As aforementioned, we designed three types of electrode configurations—emotion-specialized, attention-specialized, and general purpose configurations—with 2, 4, 6, and 8 electrodes. We employed 2-, 4-, 6-, and 8-electrode configurations, for the following reasons: (i) a greater portion of commercially available wearable EEG devices are equipped with electrodes of which the numbers range from 2 to 8, e.g., MUSE^TM^ (Interaxon Inc., Toronto, ON, Canada), Insight^TM^ (Emotiv Inc., San Francisco, CA, USA), DSI-7^TM^ (Wearable Sensing LLC, San Diego, CA, USA), Versus^TM^ (Neuro Management LLC, Scottsdale, AZ, USA), and Neuroplus^TM^ (Neuroplus Inc., Durham, NC, USA) and (ii) symmetrical electrode configurations with even numbers of electrodes were employed because electrode configurations satisfying hemispherical symmetry are much easier to implement than randomly distributed electrodes. Moreover, by assuming hemispherical symmetry, more diverse features (i.e., DASM and RASM) can be obtained. To make the electrode configurations satisfy the hemispherical symmetry condition, the selected electrodes were placed on the midline of the montage (Fz, Cz, Pz, and Oz) or existed in pairs (Fp1-Fp2, AF3-AF4, F7-F8, F3-F4, FC5-FC6, FC1-FC2, T7-T8, C3-C4, CP5-CP6, CP1-CP2, P7-P8, P3-P4, PO3-PO4, and O1-O2). 

We selected the electrode configurations that exhibited the best performance among all the possible channel combinations, with 2, 4, 6, and 8 electrodes. First, the EEG channel combinations that yielded the highest classification accuracy for the emotion dataset were selected as the emotion-specialized configurations. For a specific electrode configuration (e.g., CP1, CP2, O1, O2, F7, and F8 in the case of 6 electrodes), the highest classification accuracies achievable with the available feature combinations (e.g., combinations of multiple features selected from {6 (PSD) × 6 (channels) + 6 (DASM) × 3 (pairs) + 6 (RASM) × 3 (pairs) + 3 (HP) × 6 (channels) + 1 (SE) × 6 (channels) + 1 (HE) × 6 (channels) + 1 (KC) × 6 (channels) + 1 (HOC) × 6 (channels) + 12 (CSP) × 4 (emotion classes)} = 162 feature candidates evaluated for CP1, CP2, O1, O2, F7, and F8 channels) were calculated for each participant’s dataset. The average accuracy of classifying different emotions with the given electrode configuration was then evaluated by averaging those highest classification accuracies over all participants. This procedure was repeated for all possible electrode configurations with the designated number of electrodes, and then the electrode configuration yielding the highest average classification accuracy was reported. Similarly, the EEG channel combinations that averagely yielded the largest absolute correlation coefficients for the attention dataset were selected as the attention-specialized configurations. Finally, the EEG channel combinations that yielded the largest generalized-configuration score (GCS) were selected as the optimal configurations for both applications. This index was calculated as follows:

GCS = classification accuracy + |correlation coefficient|,(2)

where the ‘classification accuracy’ represents the average accuracy of classifying four different emotions in the emotion dataset (averaged across all participants), which ranges from 0 to 1, and the ‘|correlation coefficient|’ represents the average absolute correlation coefficient between the EEG feature and the CONC over 27 epochs in the attention dataset (averaged across all participants), which also ranges from 0 to 1. The GCS values were calculated for every possible EEG channel combination satisfying hemispherical symmetry, with 2, 4, 6, and 8 electrodes. A statistical analysis was performed via the Friedman test and Bonferroni-corrected Wilcoxon signed rank test because the testing dataset did not follow a normal distribution, as confirmed by the Kolmogorov–Smirnov test. A schematic of this procedure is shown in Figure 2c.

**Figure 3:**
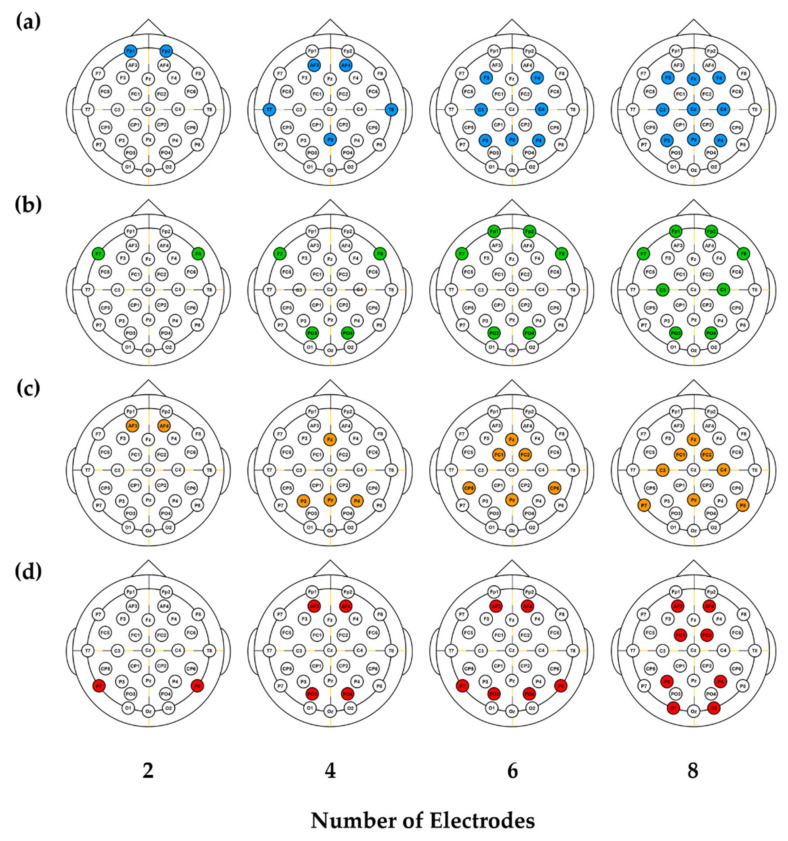
Electrode configurations adopted in this study, with different numbers of electrodes: (**a**) consumer EEG devices, (**b**) emotion-specialized design, (**c**) attention-specialized design, and (**d**) proposed design for wide pBCI applications. The consumer EEG devices adopted in this study had one more electrode than our designs, except for the device with two electrodes.

To evaluate the performance of the proposed optimal electrode configurations, the classification accuracy, correlation coefficient, and GCS were compared with those of the two application-specialized configurations and the electrode configurations of consumer EEG devices, with 2, 4, 6, and 8 electrodes. The electrode configurations of the consumer EEG devices with 2, 4, 6, and 8 electrodes were obtained from FocusBand^TM^ (T 2 Green Pty Ltd., Carrara, QLD, Australia), Insight^TM^ (Emotiv Inc., San Francisco, CA, USA), DSI-7^TM^ (Wearable Sensing LLC), and B-Alert X10^TM^ (Wearable Sensing LLC), respectively, and are shown in Figure 3a. Except for FocusBand^TM^, which was equipped with two electrodes, all the consumer EEG devices had one more than the designated number of electrodes (i.e., Insight, DSI-7^TM^, and B-Alert X10^TM^ had five, seven, and nine electrodes, respectively).

## 3. Results

### 3.1. Optimal Electrode Configurations

The electrode configurations that exhibited the best performance for emotion classification and attention estimation are presented in Figure 3b,c, respectively. The emotion-specialized electrode configurations always included F7 and F8, regardless of the number of electrodes. In addition, as the number of electrodes increased, the optimal electrode configuration could be realized simply by adding two more electrodes to the previous configuration. On the other hand, the attention-specialized electrode configurations mostly included Fz and Pz, except for the two-electrode configuration, and the electrodes were generally distributed on the central area. Similarly, the electrode configurations for the general purpose shown in Figure 3d commonly included AF3 and AF4 for 4-, 6-, and 8-electrode configurations, while the other electrodes were generally distributed on the parietal area. 

The use of different classifiers (LDA and DT) resulted in somewhat different optimal electrode configurations compared to those obtained using SVM, but the overall trend was similar. For example, the emotion-specialized electrode configurations commonly shared F7 and F8 regardless of the numbers of electrodes (see Figure S1 in the Supplementary Material). Likewise, the general purpose electrode configurations commonly shared AF3 and AF4 regardless of the numbers of electrodes.

### 3.2. Performance Comparison

**Figure 4:**
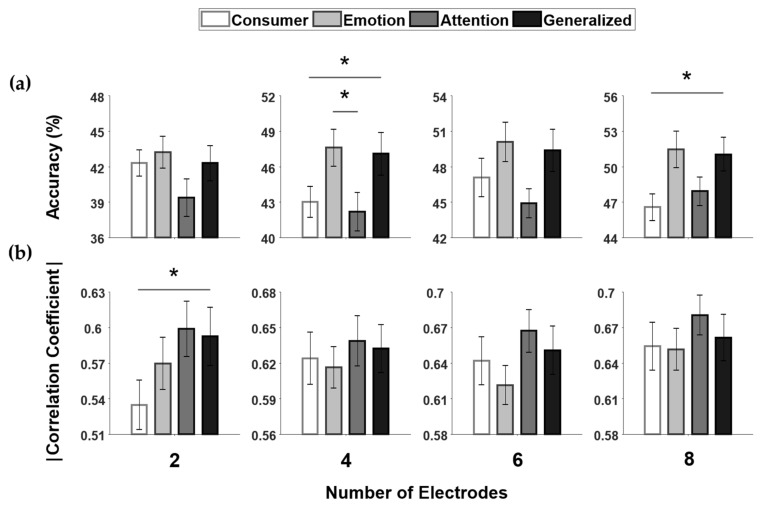
Comparison of classification and estimation performances among the consumer EEG device (denoted as “Consumer”), emotion-specialized device (denoted as “Emotion”), attention-specialized device (denoted as “Attention”), and general purpose device (denoted as “Generalized”), with different numbers of electrodes. The error bars indicate the standard errors across participants. (**a**) Classification accuracy calculated using the emotion dataset and (**b**) absolute correlation coefficient calculated using the attention dataset.

The performance of emotion classification and attention estimation was compared among the electrode configurations shown in Figure 4. Figure 4a shows a comparison of the average accuracy of four-class emotion classification among the four types of electrode configurations. The emotion-specialized electrode configuration consistently exhibited the best performance, followed by the general purpose configuration, regardless of the number of electrodes. The Friedman test indicated statistical significance for the 4-, 6-, and 8-electrode cases (χ^2^ = 2.14, *p* = 0.544; χ^2^ = 10.13, *p* < 0.05; χ^2^ = 8.75, *p* < 0.05; and χ^2^ = 12.53, *p* < 0.01 for 2, 4, 6, and 8 electrodes, respectively). The Wilcoxon signed rank post-hoc test with Bonferroni correction showed that the classification accuracy for the general purpose electrode configuration was significantly higher than those for consumer devices for 4-, and 8-electrode cases (Bonferroni-corrected *p* < 0.05 in both cases) and the classification accuracy for the emotion-specialized electrode configuration was significantly higher than that for the attention-specialized electrode configuration for 4-electrode case (Bonferroni-corrected *p* < 0.05). Additionally, the precision and recall for emotion classification were compared among the four types of electrode configurations, as presented in Figure S2 in the Supplementary Material. In most cases, the emotion-specialized and general purpose electrode configurations showed relatively better performances than the consumer EEG devices and attention-specialized electrode configurations.

Figure 4b shows the average absolute correlation coefficients between the EEG measure and the CONC for the four types of electrode configurations. The attention-specialized configuration exhibited the best performance, followed by the general purpose configuration, regardless of the number of electrodes. The Friedman test reported statistical significance for the 2-, 6-, and 8-electrode cases (χ^2^ = 12.72, *p* < 0.01; χ^2^ = 2.04, *p* = 0.564; χ^2^ = 12.53, *p* < 0.01; and χ^2^ = 11.61, *p* < 0.01 for 2, 4, 6, and 8 electrodes, respectively). The Wilcoxon signed rank post-hoc test with Bonferroni correction showed that the absolute correlation coefficient for the general purpose electrode configuration was significantly higher than that for the consumer device with two electrodes (Bonferroni-corrected *p* < 0.01). Notably, the general purpose electrode configurations always exhibited better performance than the electrode configurations of consumer EEG devices, regardless of the number of channels, even though the proposed general purpose configurations had lesser electrodes than the configurations of the consumer EEG devices.

**Figure 5:**
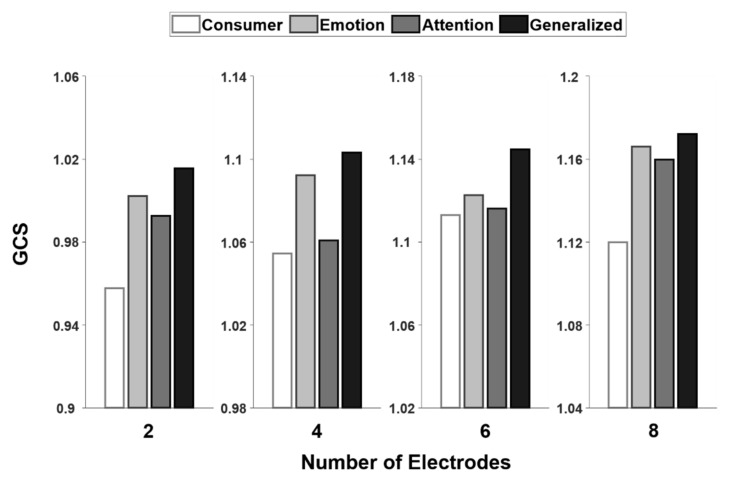
Comparison of generalized-configuration score (GCS) values among the consumer EEG device (denoted as “Consumer”), emotion-specialized device (denoted as “Emotion”), attention-specialized device (denoted as “Attention”), and general purpose device (denoted as “Generalized”), with different numbers of electrodes. There was only a single GCS value for each case.

**Figure 6:**
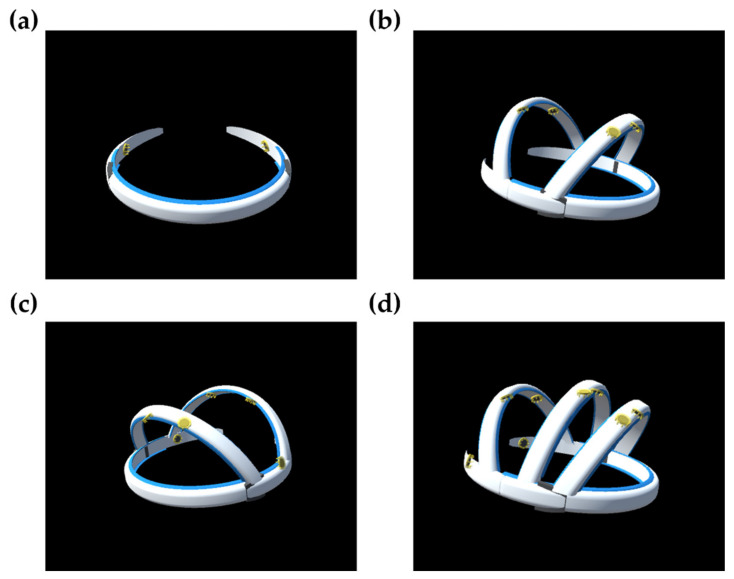
3D rendering images of custom-designed wearable EEG devices with general purpose electrode configurations with (**a**) two, (**b**) four, (**c**), six, and (**d**) eight electrodes.

Figure 5 shows comparisons of the GCS values among the four types of electrode configurations, with different numbers of electrodes. Among the four configurations, the general purpose configuration exhibited the highest GCS regardless of the number of electrodes. A statistical analysis was not possible here, because only a single GCS value was obtained for each case. Additionally, we virtually designed wearable EEG devices with the proposed general purpose electrode configurations, with 2, 4, 6, and 8 electrodes. Three-dimensional (3D) rendered images of the custom-designed wearable EEG devices are shown in Figure 6. The devices were assumed to have comb-type dry EEG electrodes [26]. The EEG devices were designed to be headset-type devices.

The performance of emotion classification was compared among the electrode configurations determined using LDA and DT, of which the results are shown in Figures S3a and S4a in the Supplementary Material, respectively. In line with the SVM results, the emotion-specialized electrode configuration consistently exhibited the best performance, followed by the general purpose configuration, regardless of the number of electrodes. The Friedman test indicated statistical significance for the 2-, 6-, and 8-electrode cases for LDA, and 4-, and 6-electrode cases for DT (χ^2^ = 8.01, *p* < 0.05; χ^2^ = 9.04, *p* < 0.05; χ^2^ = 16.06, *p* < 0.005; χ^2^ = 17.13, *p* < 0.001; and χ^2^ = 14.13, *p* < 0.005 for 2, 6, and 8 electrodes with LDA and 4, and 6 electrodes with DT, respectively). The Wilcoxon signed rank post-hoc test with Bonferroni correction showed that the classification accuracy for the general purpose electrode configuration and emotion-specialized electrode configuration were significantly higher than that for consumer devices for the 8-electrode case with LDA (Bonferroni-corrected *p* < 0.05 in both cases). Moreover, the classification accuracies for the emotion-specialized electrode configuration and general purpose electrode configuration were significantly higher than those for the attention-specialized electrode configurations for 4-, and 6-electrode cases with DT (Bonferroni-corrected *p* < 0.05 in all cases; especially the Bonferroni-corrected *p* < 0.01 between emotion-specialized electrode configuration and attention-specialized electrode configuration).

Figures S3b and S4b show the average absolute correlation coefficients between the EEG measure and the CONC for the four types of electrode configurations determined using LDA and DT. In line with the SVM results, the attention-specialized configuration exhibited the best performance, followed by the general purpose configuration, regardless of the number of electrodes. The Friedman test reported statistical significance for the 2-, and 8-electrode cases with DT (χ^2^ = 13.56, *p* < 0.005; and χ^2^ = 12.21, *p* = 0.01 for 2, and 8 electrodes, respectively). The Wilcoxon signed rank post-hoc test with Bonferroni correction showed that the absolute correlation coefficient for the consumer device was significantly lower than those for the three other types of electrode configurations for 2-electrode case with DT (Bonferroni-corrected *p* < 0.05 in all cases), and the absolute correlation coefficient for the attention-specialized electrode configuration was significantly higher than that for the consumer device for the 8-electrode case with DT (Bonferroni-corrected *p* < 0.05).

Figures S5 and S6 show the comparisons of the GCS values among the four types of electrode configurations determined based on LDA and DT, respectively, with different numbers of electrodes. In line with the SVM results, the general purpose configuration exhibited the highest GCS regardless of the number of electrodes.

## 4. Discussion

In the present study, we investigated specific application-specialized electrode configurations that maximize the classification accuracy of emotion recognition or the correlation coefficient in attention estimation. We then searched for general purpose electrode configurations that can be employed in both applications, with slightly lower accuracies than those of the specific application-specialized configurations but higher accuracies than those of the commercial EEG devices. The general purpose electrode configurations always exhibited better performance than the consumer EEG devices, regardless of the number of electrodes, even though the number of electrodes in the consumer EEG devices was equal to or greater than that in the newly designed devices. These results suggest that an EEG device with an electrode configuration optimized based on reliable datasets can potentially exhibit better performance than an EEG device with a larger number of electrodes.

There have been some previous studies that investigated the optimal electrode placements for specific EEG applications as listed in Table 2 [39,40,41,42]. However, all the previous studies determined the electrode configurations only for a single specific application and did not propose general purpose electrode configurations. In addition, the EEG datasets employed in this study were recorded from more numbers of channels than those employed in the previous studies, allowing for the performance comparison among more diverse electrode combinations. Lastly, we proposed optimal electrode configurations with various numbers of electrodes (2, 4, 6, and 8 electrodes), while the previous studies presented their optimal electrode configurations with limited numbers of electrodes.

Each application-specialized and general purpose electrode configuration frequently included common electrodes across the number of electrodes. For example, emotion-specialized electrode configurations included F7 and F8 across all numbers of electrodes, and electrode configuration for general purpose mostly included AF3 and AF4, except for the 2-electrode configuration. Indeed, it has been reported that EEG data recorded from the frontal area contain informative signals associated with various mental states of humans, including emotion and attention [43,44,45]. Similarly, attention-specialized electrode configurations mostly included Fz and Cz, except for the 2-electrode configuration. It might imply that Fz and Pz play a crucial role in attention estimation, as frequently reported in the previous literature [46,47,48]. In addition, many electrodes included in application-specialized or general purpose electrode configurations were distributed in the parietal area, which is in line with the previous literatures reporting that the brain activities recorded in this area reflect a wide range of mental states [49,50,51]. 

In the present study, we only investigated electrode configurations optimized for emotion recognition and attention monitoring applications in specific paradigms (emotion modulation via audiovisual stimuli and attention estimation under the d2 task). Further studies need to be performed to find more generalized electrode configurations that can cover more mental state modulation paradigms and elevate the performance of other pBCI applications based on the estimation of user’s mental workload, relaxation, and boredom. Please note that the ultimate goal of the present study was to present a detailed procedure for designing wearable EEG devices that can maximize the overall performance of specific pBCI applications by utilizing EEG databases associated with these applications. The optimal electrode configurations may vary depending on the employed datasets, paradigms, and EEG indices. Designing a new general purpose EEG device that can yield improved performance for a wider range of pBCI applications is an interesting topic that we wish to pursue in the future studies.

Recently, the remarkable advancements in system-on-chip (SoC) technology have led to the release of various consumer EEG devices that have potential to be applied in the real world. If the newly released consumer EEG devices are integrated with advanced signal-processing technologies, the use of pBCIs in daily life will be feasible [52]. Apart from the four consumer EEG devices tested in the present study, numerous wearable EEG devices have been released on the market, targeting various pBCI applications. MyndBand^TM^ (Myndplay Ltd., London, UK), Versus^TM^ (Neuro Management LLC), and Neuroplus^TM^ (Neuroplus Inc.) were released to improve the mental concentration of users. DSI VR300^TM^ (Wearable Sensing LLC), NeuroSky^TM^ (NeuroSky Inc., San Jose, CA, USA), and 4D Force^TM^ (4DForce GmbH, Meiningen, Germany) were developed for neuroeducation. BrainLink^TM^ (Macrotellect Ltd., Shenzhen, Nanshan District, China) and MUSE^TM^ (Interaxon Inc., Toronto, ON, Canada) were developed for meditation and stress management. Additionally, several devices were developed for sleep management, including Kokoon^TM^ (Kokoon Technology, London, UK), Bedside Sleep Manager^TM^ (Zeo Inc., Newton, MA, USA), and Dreem^TM^ (Dreem, Paris, France) [53,54]. SmartCap^TM^ (SmartCap Technologies, Milton, QLD, Australia) was developed to monitor the fatigue levels of workers or the drowsiness of drivers to prevent accidents. Agile-10^TM^ (Cognionics Inc., San Diego, CA, USA), Enobio^®^ (Neuroelectrics, Barcelona, Spain), and g.MOBIlab+^TM^ (g.tec Medical Engineering GmbH, Schiedlberg, Austria) were developed to aid out-of-the-lab applications with research-level signal quality. However, in most of the EEG devices on the market that is targeting a specific application, the procedure for designing the electrode configurations still remains unclear. For the practical use of pBCI applications in daily life, it is essential to develop EEG devices with an electrode configuration optimized using reliable EEG datasets. In summary, this study provided insights regarding the design of the optimal electrode configuration for general purpose consumer EEG devices.

## Figures and Tables

**Table 1:** List of electroencephalogram (EEG) features evaluated in the present study.

Feature	Mathematical Expression
Power Spectral Density (PSD) ^1^	$\frac{1}{N}\sum_{n=1}^{N}x\left(n\right)e^{-i2\pi fn/N}\;$
Differential Asymmetry (DASM)	Difference between PSDs of interhemispheric electrode pairs
Rational Asymmetry (RASM)	Ratio between PSDs of interhemispheric electrode pairs
Hjorth Parameters ^2^ [30]	$\mathrm{Activity}\left(x\right)=\frac{1}{N}\sum_{i=1}^{N}{\left(x_{i}-\mu_{x}\right)}^{2}$
	$\mathrm{Mobility}\left(x\right)=\sqrt{\frac{\sigma (x\prime )}{\sigma \left(x\right)}}$
	$\mathrm{Complexity}\left(x\right)=\frac{Mobility\left(x\prime \right)}{Mobility\left(x\right)}$
Shannon Entropy ^3^ [31]	$-\sum_{i=1}^{N}p(x_{i})\ln p\left(x_{i}\right)\;,\;where\;\sum_{i=1}^{N}p(x_{i})=1$
Hurst Exponent ^4^ [32]	$\log\left(R/S\right)/\log\left(N\right)$
Kolmogorov Complexity ^5^ [33]	c(*n*)/b(*n*)
Higher-order Cumulants ^6^ [34]	$E\left[X\left(k\right)X\left(k+m\right)X\left(k+n\right)\right]$
Common Spatial Pattern ^7^ [35]	$f_{p}=\log\frac{var\left(Z_{p}\right)}{\sum_{i=1}^{2m}var\left(Z_{i}\right)}$, where *p* = 1 to 2*m*

^1^*x* represents the EEG time-series data and *N* indicates the length of data. ^2^ *x* represents the EEG-time series data, $\mu_{x}$ represents the mean of *x*, *x*’ represents the derivative of *x*, and σ(x) represents the standard deviation of *x*. ^3^ ***x*** represents the EEG time-series data. ^4^ *N* is the length of the sample of data, *R* is the difference between the maximum deviation from the mean and the minimum deviation from the mean, and *S* is the standard deviation. ^5^ *n* is the length of the time series data and *b*(*n*) is the ratio between *n* and log(*n*). ^6^ *X*(*n*) is the Fourier transform of the signal *x*(*n*) and *E*[·] stands for the expectation operation. ^7^ *Z_p_* indicate the spatially filtered EEG signals associated with the largest *p* eigenvalues of sum of covariance matrix. More details can be found in [35]. Note that the value of *p* was set to 2 in this study.

**Table 2:** Comparison with previous literatures on optimal electrode configuration for EEG applications.

Study	Total Number of Electrodes	Number of Electrodes of Proposed Design	Applications
Ganguly et al. [39]	11	1	Emotion Classification
Siamaknejad et al. [40]	20	1	Attention Estimation
Abdullah et al. [41]	8	2, 4	Biometric Recognition
Yang et al. [42]	20	4	Biometric Recognition
Present Study	32	2, 4, 6, 8	Emotion Classification Attention Estimation

## Supplementary Materials

The following are available online at https://www.mdpi.com/1424-8220/20/16/4572/s1.
