# Supplementary material for: Design of Wearable EEG Devices Specialized for Passive Brain–Computer Interface Applications
Source: Sensors (Basel). 2020 Aug 14;20(16):4572. doi: 10.3390/s20164572 (PMC7472161; doi:10.3390/s20164572)
Supplement: Supplementary file 1 [file sensors-20-04572-s001.pdf]

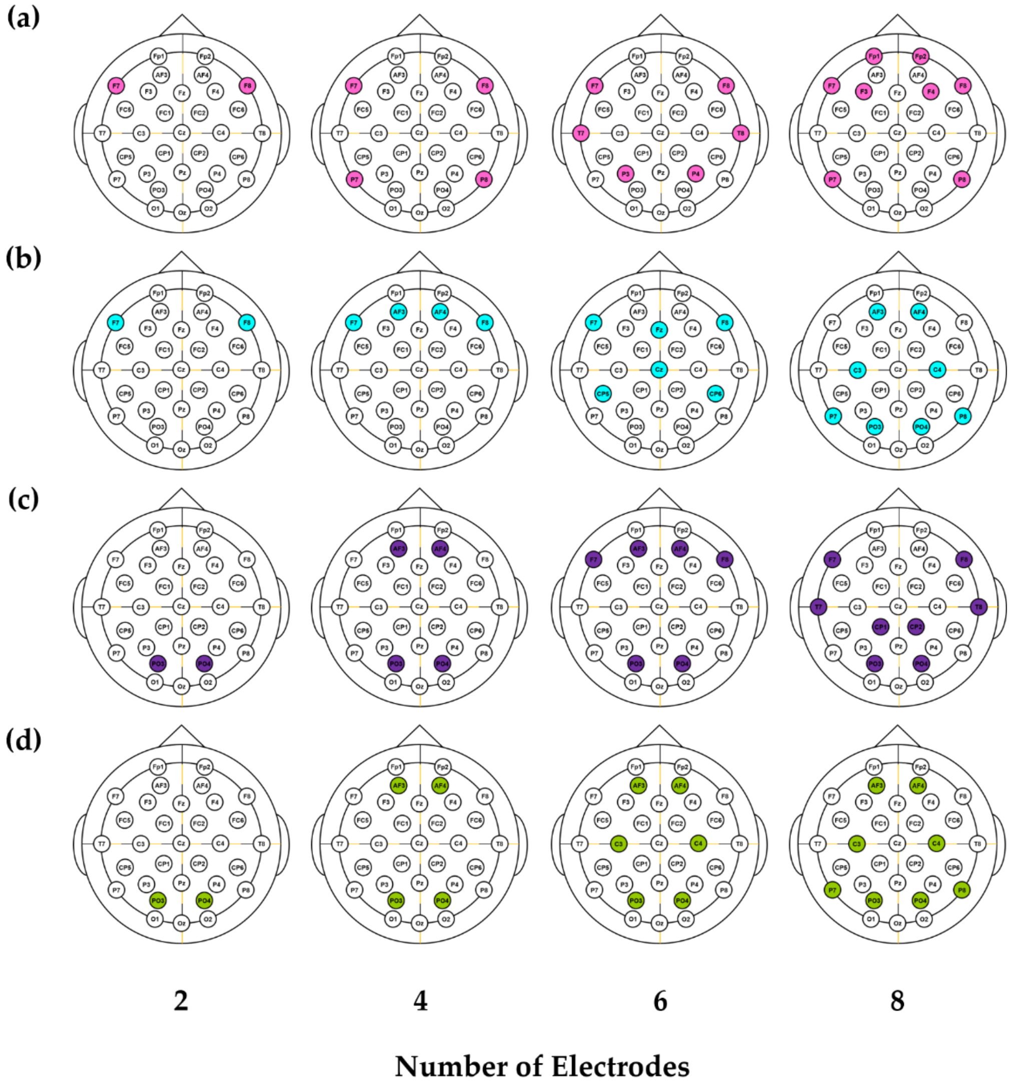

Figure S1. Optimal electrode configurations obtained using LDA and DT classifiers, with different numbers of electrodes: (a) LDA-based emotion-specialized design, (b) LDA-based general-purpose design, (c) DT-based emotion-specialized design, and (d) DT-based general-purpose design.

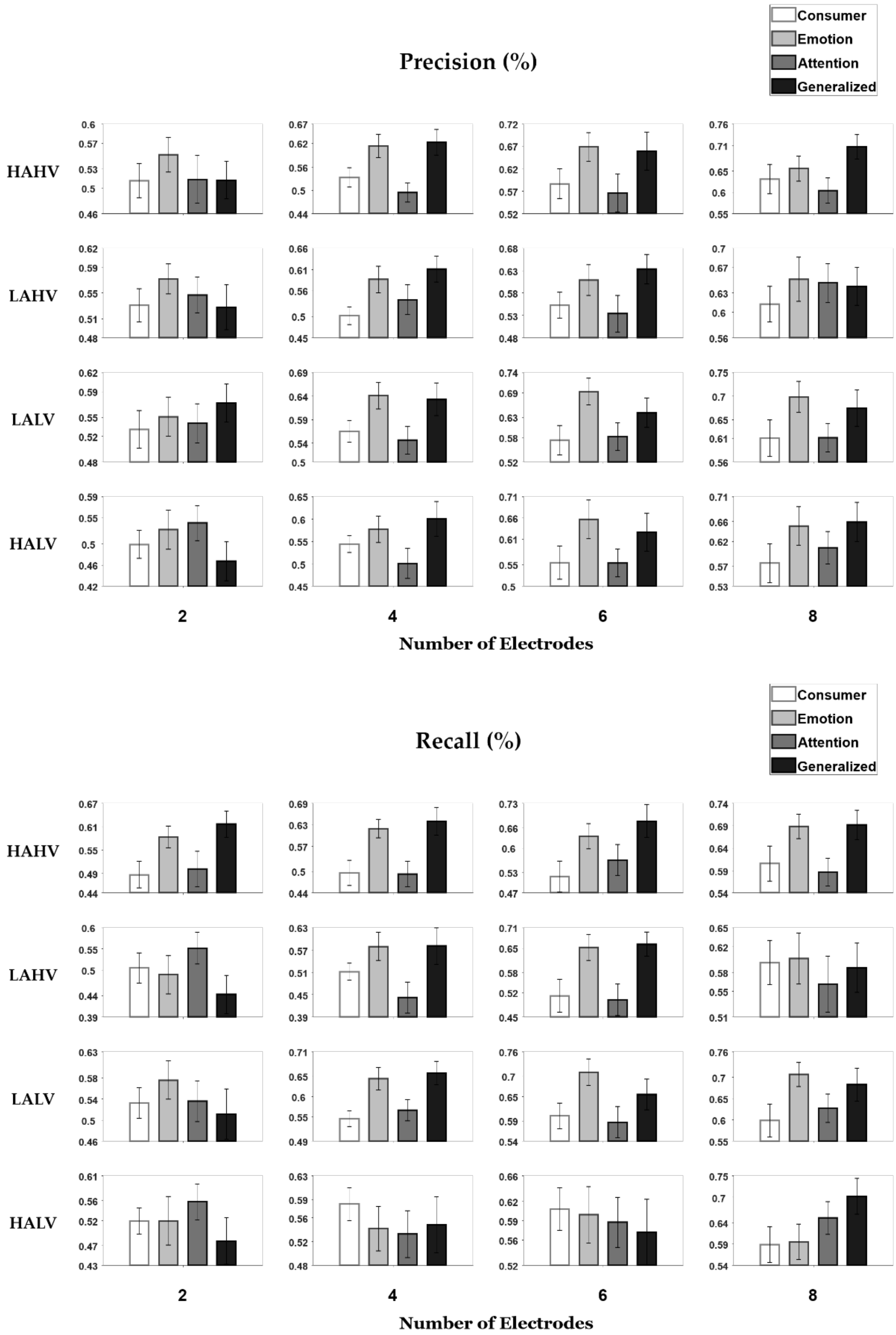

Figure S2. Precision and recall of each emotional state for the four types of electrode configurations, with respect to the number of electrodes. Precision and recall were computed with the following equations: Precision = TP/(TP+FP); Recall = TP/(TP+FN), where TP, FP and FN represent true positive, false positive, and false negative, respectively.

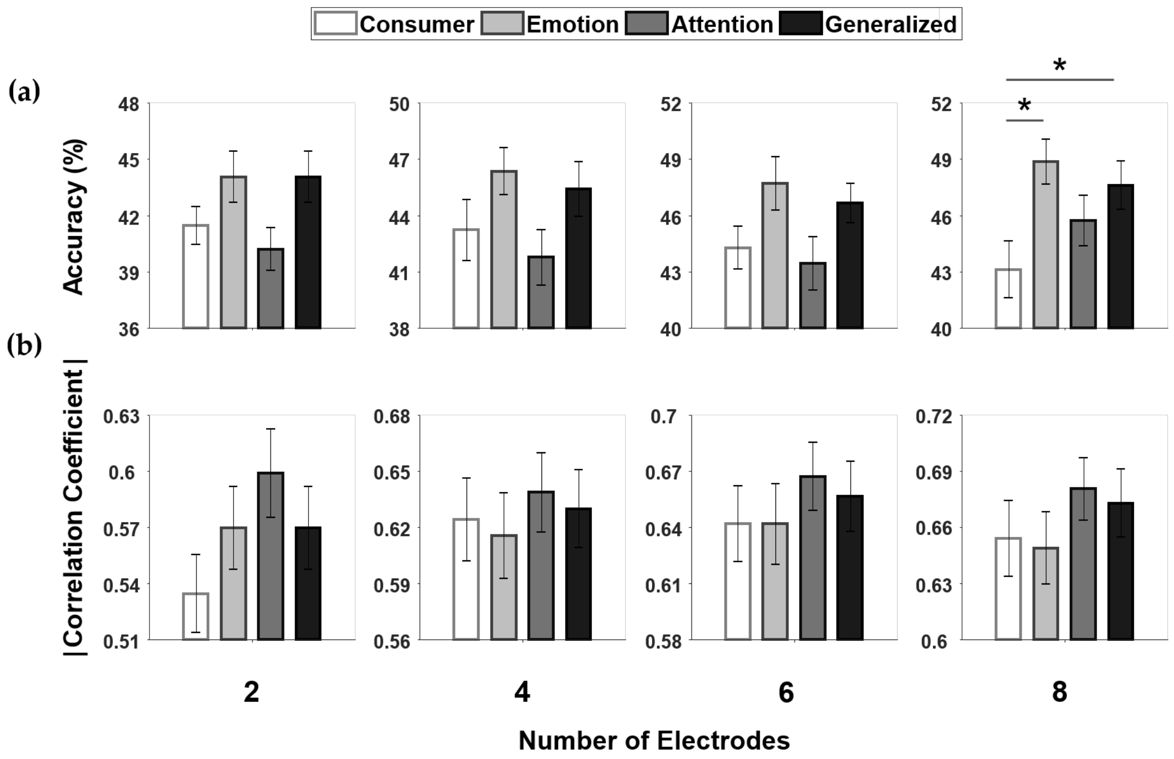

Figure S3. Comparison of LDA-based classification and estimation performances among the consumer EEG device (denoted as "Consumer"), emotion-specialized device (denoted as "Emotion"), attention-specialized device (denoted as "Attention"), and general-purpose device (denoted as "Generalized"), with different numbers of electrodes. The error bars indicate the standard errors across participants. (a) Classification accuracy calculated using emotion dataset; (b) absolute correlation coefficient calculated using attention dataset.

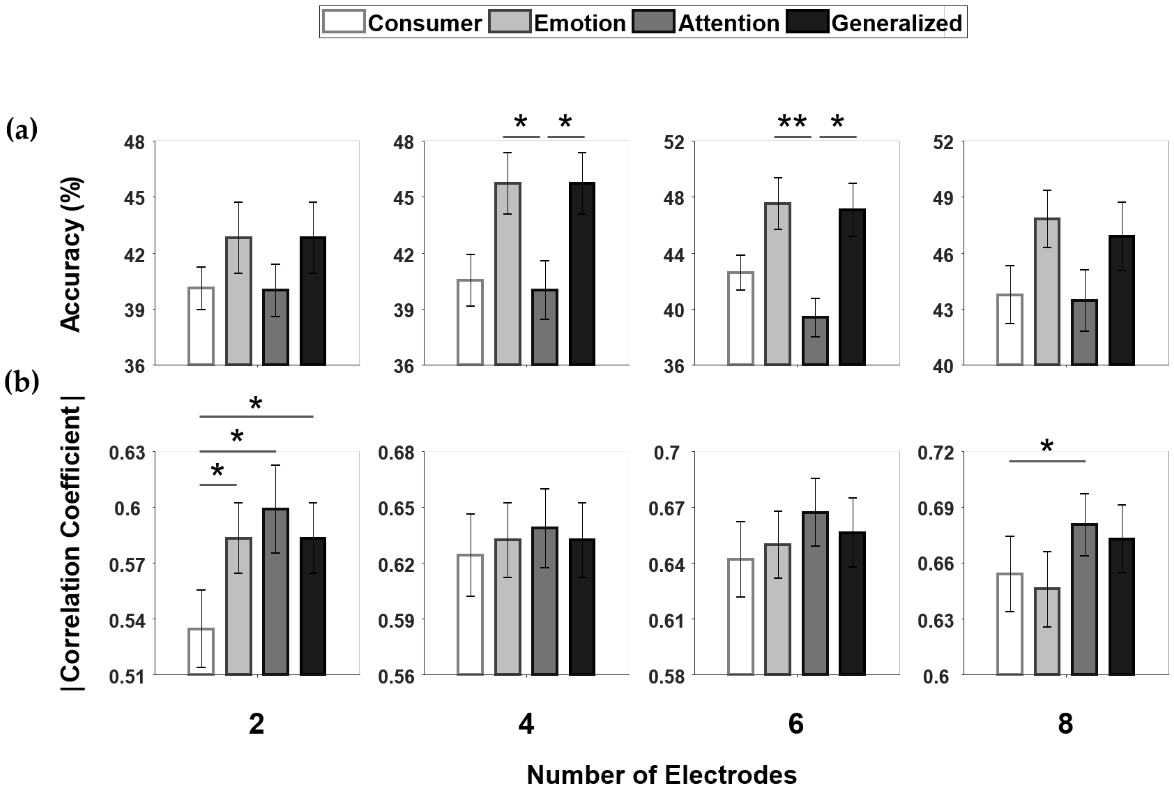

Figure S4. Comparison of DT-based classification and estimation performances among the consumer EEG device (denoted as "Consumer"), emotion-specialized device (denoted as "Emotion"), attention-specialized device (denoted as "Attention"), and general-purpose device (denoted as "Generalized"), with different numbers of electrodes. The error bars indicate the standard errors across participants. (a) Classification accuracy calculated using emotion dataset; (b) absolute correlation coefficient calculated using attention dataset.

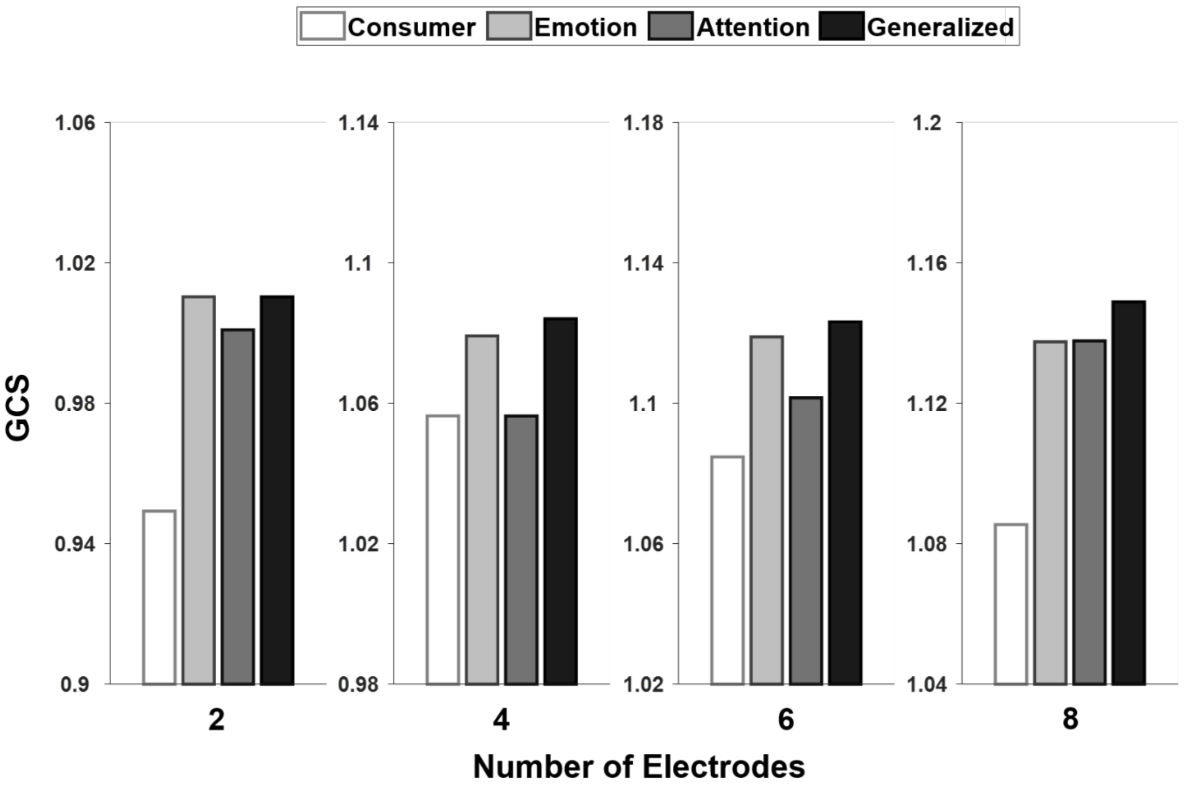

Figure S5. Comparison of LDA-based GCS values among the consumer EEG device (denoted as "Consumer"), emotion-specialized device (denoted as "Emotion"), attention-specialized device (denoted as "Attention"), and general-purpose device (denoted as "Generalized"), with different numbers of electrodes. There was only a single GCS value for each case.

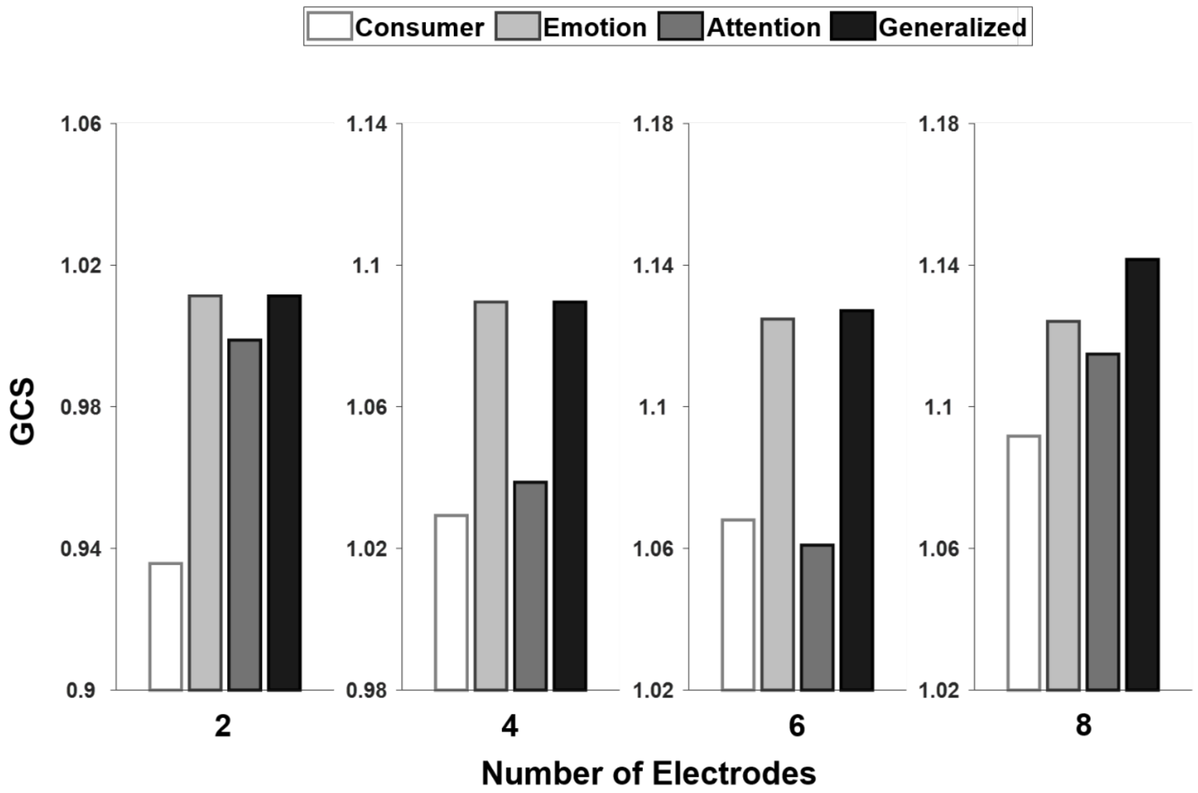

Figure S6. Comparison of DT-based GCS values among the consumer EEG device (denoted as "Consumer"), emotion-specialized device (denoted as "Emotion"), attention-specialized device (denoted as "Attention"), and general-purpose device (denoted as "Generalized"), with different numbers of electrodes. There was only a single GCS value for each case.
